# *Staphylococcus aureus* Synergized with *Candida albicans* to Increase the Pathogenesis and Drug Resistance in Cutaneous Abscess and Peritonitis Murine Models

**DOI:** 10.3390/pathogens10081036

**Published:** 2021-08-16

**Authors:** Yao Hu, Yulong Niu, Xingchen Ye, Chengguang Zhu, Ting Tong, Yujie Zhou, Xuedong Zhou, Lei Cheng, Biao Ren

**Affiliations:** 1 State Key Laboratory of Oral Diseases, West China School of Stomatology, National Clinical Research Center for Oral Diseases, Sichuan University, Chengdu 610064, China; huyao93@stu.scu.edu.cn (Y.H.); 2017151651111@stu.scu.edu.cn (X.Y.); 2012181643027@stu.scu.edu.cn (C.Z.); 2017224035075@stu.scu.edu.cn (T.T.); 2018324035021@stu.scu.edu.cn (Y.Z.); 2 Key Laboratory of Bio-Resources and Eco-Environment of Ministry of Education, College of Life Sciences, Sichuan University, Chengdu 610041, China; yulong.niu@scu.edu.cn

**Keywords:** *Candida albicans*, *Staphylococcus aureus*, synergistic effect, virulence, drug resistance

## Abstract

The mixed species of *Staphylococcus aureus* and *Candida albicans* can cause infections on skin, mucosa or bloodstream; however, mechanisms of their cross-kingdom interactions related to pathogenesis and drug resistance are still not clear. Here an increase of *S. aureus* proliferation and biofilm formation was observed in *S. aureus* and *C. albicans* dual-species culture, and the synergistic pathogenic effect was then confirmed in both local (cutaneous abscess) and systemic infection (peritonitis) murine models. According to the transcriptome analysis of the dual-species culture, virulence factors of *S. aureus* were significantly upregulated. Surprisingly, the beta-lactams and vancomycin-resistant genes in *S. aureus* as well as azole-resistant genes in *C. albicans* were also significantly increased. The synergistic effects on drug resistance to both antibacterial and antifungal agents were further proved both in vitro and in cutaneous abscess and peritonitis murine models treated by methicillin, vancomycin and fluconazole. The synergistic interactions between *S. aureus* and *C. albicans* on pathogenesis and drug resistance highlight the importance of targeting the microbial interactions in polyspecies-associated infections.

## 1. Introduction

The interactions between microbial species can impact pathogenic behaviors such as proliferation, virulence, and antibiotic tolerance during polyspecies-related infections [1,2,3,4]. The resistance of pathogenic species to antibiotics has already become one of the greatest challenges in global health [5]. Infections caused by drug-resistant microorganisms are associated with increased mortality as well as economic burden. It is estimated that by 2050, antibiotic-resistant organisms will cause 300 million premature deaths [6,7]. Meanwhile, the increase of virulence of polyspecies-related infections caused by interspecies interactions can lead to poor prognosis of patients [8,9].

*Staphylococcus aureus* and *Candida albicans* have been recognized as parts of the normal microbiota of human bodies, but they can cause mixed-species infections on skin, mucosa or in the bloodstream [8,10,11,12,13]. *S. aureus* is recognized as the third most common organism co-isolated with *C. albicans* in nosocomial *C. albicans* blood system infections [14,15,16]. In systemic candidiasis, the common detection rate of *S. aureus* and *C. albicans* is 20% [14,16,17,18,19,20,21], while in catheter infections, burn wound infections, denture stomatitis, as well as peri-implantitis, the co-detection of *S. aureus* and *C. albicans* is also common [10,11,12,22,23]. Synergistic pathogenic effects between *S. aureus* and *C. albicans* have also been observed [24,25,26,27]; for example, intraperitoneally injecting sub-lethal doses of *S. aureus* and *C. albicans* increased mice mortality from 0% to 40–100%, while reducing its median effective dose to 1/70,000 [25]. In a *Galleria mellonella* larvae testing model, co-infection by *S. aureus* and *C. albicans* significantly reduced the larval survival rate, with a higher density of *S. aureus* [28]. In addition to synergistic pathogenesis, *S. aureus* and *C. albicans* can mutually promote drug resistance. *C. albicans* can increase the resistance of *S. aureus* to vancomycin about 100-fold [24], while *C. albicans* and *S. aureus* dual-species infection was shown to be concomitantly desensitized to miconazole treatment in a *Galleria mellonella* model [29].

The farnesol and prostaglandin E_2_ secreted by *C. albicans* were thought to enhance biofilm formation of *S. aureus* [17]. Additionally, *S. aureus* virulence might be augmented by *C. albicans* through the accessory gene regulator system [30,31]. As for mutually elevated antibiotic tolerance, the extracellular matrix is considered as the key factor. Harriott et al. believe that *S. aureus* may be coated in the matrix secreted by *C. albicans*, which could be the reason for increasing drug resistance [24]. They also investigated the role of adhering ability in promoting the resistance of *S. aureus* to vancomycin, confirming that only mutants that can adhere to the abiotic surface can induce *S. aureus* vancomycin resistance, regardless of the presence of hyphae [32]. A *C. albicans*-secreted β-1,3-glucan cell wall component was identified as the key matrix constituent providing the bacterium with enhanced drug tolerance [27]. However, how *C. albicans* regulates the gene expression related to the virulence and antibacterial drug resistance of *S. aureus*, and how *S. aureus* influences the antifungal drug resistant gene expression of *C. albicans*, as well as their synergistic mechanisms and the resistant effects to antibacterial and antifungal drugs in both local and systemic infectious models, are still not clear. Here we investigated the synergistic pathogenesis and drug resistance in cutaneous abscess and peritonitis murine models and identified mechanisms through the transcriptomic analysis of co-cultures to highlight the importance of interspecies interactions in polyspecies infections.

## 2. Results

### 2.1. C. albicans Promoted Proliferation and Biofilm-Formation of S. aureus

**Figure 1:**
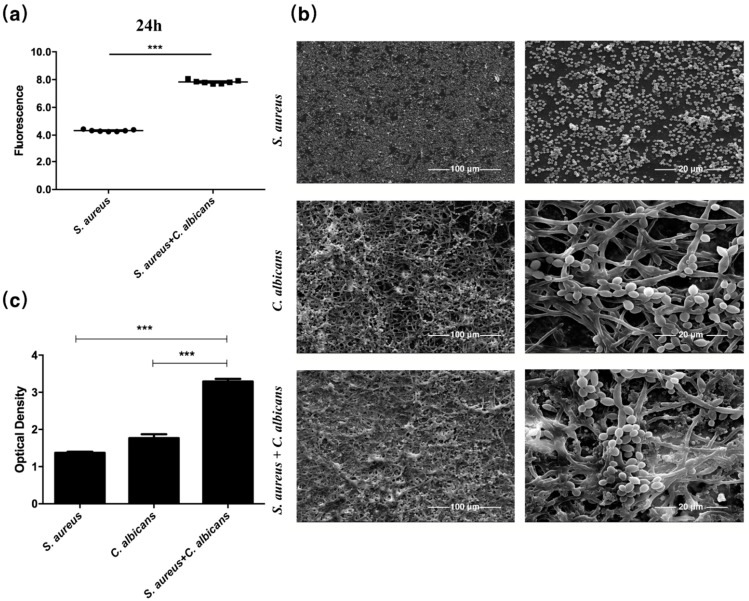
*C. albicans* promoted proliferation and biofilm formation of *S. aureus*. (**a**) GFP expressing *S. aureus* was co-cultured with *C. albicans*. GFP fluorescence was detected at 24 h (*n* = 7). (**b**) Scanning electron micrographs of single-species or dual-species biofilms. (**c**) Biofilm thickness of single-species or dual-species biofilms determined by crystal violet staining (*n* = 5). ***: *p* < 0.001.

A GFP expressing *S. aureus* strain was employed for assessing the effect of *C. albicans* on the proliferation of *S. aureus*. A significant increase of GFP fluorescence was detected in strains co-cultured with *C. albicans* both at 14 h and 24 h (Figure 1a and Figure S1). The single-species or dual-species biofilms of *C. albicans* and *S. aureus* were observed by SEM, and the biofilm biomass was determined by crystal violet staining. The combination of *S. aureus* and *C. albicans* significantly increased the biofilm formation and the extracellular matrix (Figure 1b), and the biofilm biomass was extensively elevated compared to single-species biofilms (Figure 1c).

### 2.2. C. albicans and S. aureus Synergistically Increased the Pathogenicity in Cutaneous Abscess and Peritonitis Murine Models

**Figure 2:**
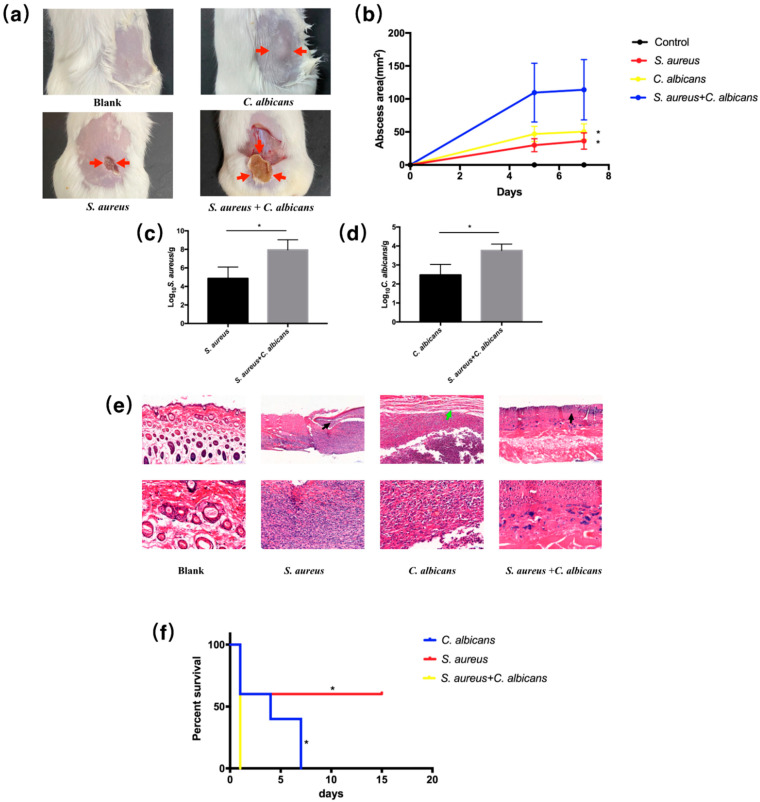
*C. albicans* and *S. aureus* synergistically increased pathogenicity in local infection murine model and in systemic infection murine model. (**a**) Images of infected mice skin after 7 day inoculation of normal saline, *C. albicans, S. aureus* or both organisms. Cutaneous abscess is indicated by red arrows. (**b**) Abscess area measured at day 0, 5 and 7 (*n* = 4); *: *p* < 0.05 compared to the *S. aureus* + *C. albicans* group. (**c**) *S. aureus* burdens obtained from the infected skin after 7 day inoculation (*n* = 3). *: *p* < 0.05. (**d**) *C. albicans* burdens obtained from the infected skin after 7 day inoculation (*n* = 3). *: *p* < 0.05. (**e**) Representative histological images the mice skin. Necrosis is indicated by black arrows and subcutaneous nodule is indicated by green arrows. (**f**) Survival curves of mice intraperitoneally infected with *C. albicans, S. aureus* or both organisms (*n* = 5); *: *p* < 0.05 compared to the *S. aureus* + *C. albicans* group.

Both superficial dermonecrosis and subcutaneous abscess could be seen from the pathogen-infected mice, as indicated by red arrows in Figure 2a, and the dual-species treated mice displayed increased abscess area and microbial burdens of both microorganisms (Figure 2b–d). The histological analysis showed more extensive skin necrosis in the mice infected by the dual species (Figure 2e). For the peritonitis model, mice mortality was monitored for 15 days after intraperitoneally inoculation with *S. aureus* and *C. albicans* and their combinations. Simultaneous injection of *C. albicans* and *S. aureus* significantly decreased the survival rate of mice compared with single-species treated mice (Figure 2f).

### 2.3. Staphylococcal Genes Related to Virulence Were Activated by C. albicans

**Figure 3:**
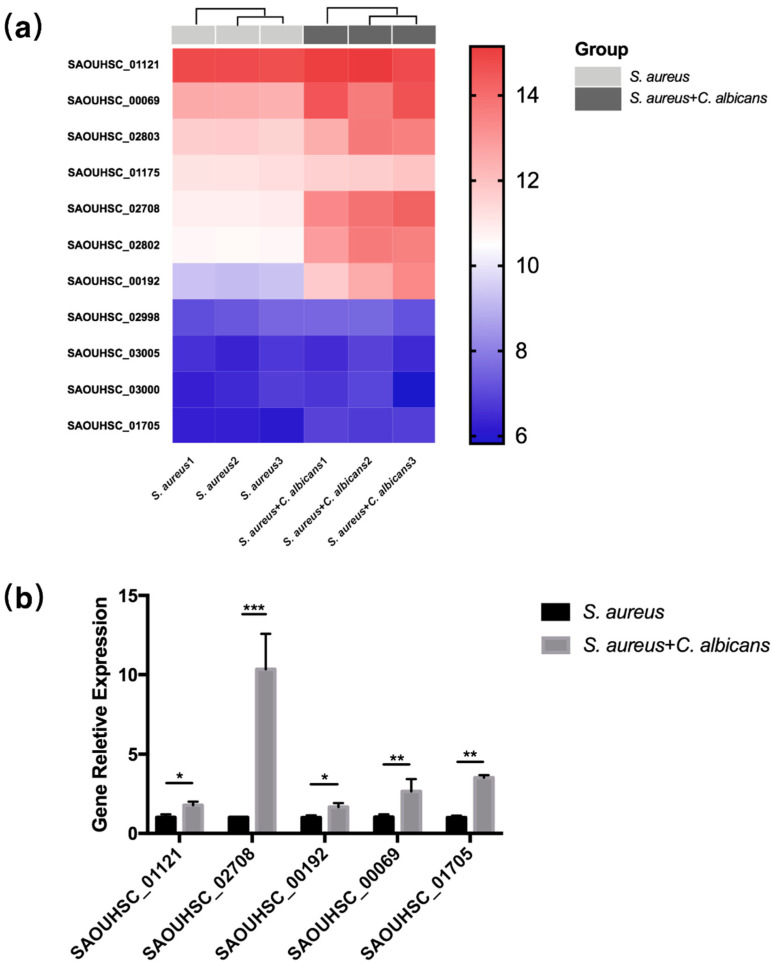
Staphylococcal genes related to virulence were activated by *C. albicans*. (**a**) Heat map of differentially expressed staphylococcal virulence genes (*n* = 3). (**b**) Relative expression of virulence factors in *S. aureus* or co-cultured *S. aureus* measured by RT-qPCR (*n* = 3). All gene expression levels were normalized by 16 s rRNA gene expression. *: *p* < 0.05; **: *p* < 0.01; ***: *p* < 0.001.

Transcriptome analysis was then conducted to explore the mechanisms behind the synergistic effect of *C. albicans* and *S. aureus*. Among the significantly enriched pathways of *C. albicans* and *S. aureus* co-cultured samples, the pathways “*Staphylococcus aureus* infection” as well as “Quorum sensing” were remarkably changed (Figure S2), and the terms consisting of “Toxin activity” were also enriched (Figure S3). Most of the virulence factors of *S. aureus* were significantly up-regulated in the co-cultured group (Figure 3a), including gamma-hemolysin (SAOUHSC_02708), staphylocoagulase (SAOUHSC_00192), staphylococcal protein A (SAOUHSC_00069), enterotoxin family protein (SAOUHSC_01705), alpha-hemolysin (SAOUHSC_01121), nucleases, intercellular adhesion proteins and fibronectin-binding proteins. The up-regulated virulence factors were further confirmed by RT-qPCR analysis (Figure 3b).

### 2.4. C. albicans Augmented Beta-Lactams and Vancomycin-Resistant Gene Expression in S. aureus

**Figure 4:**
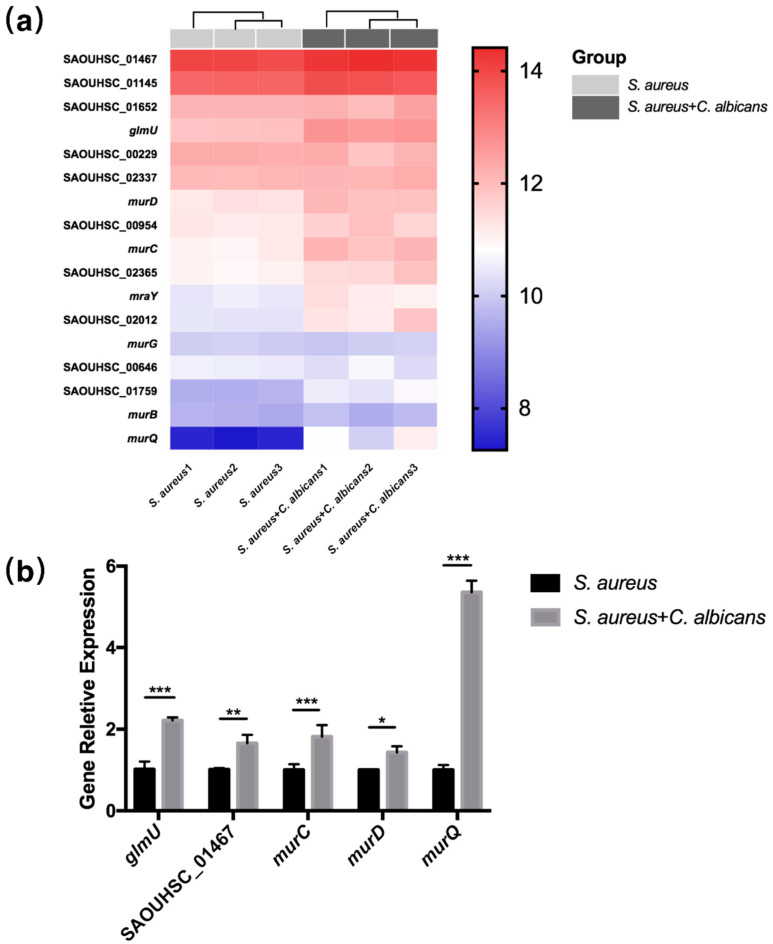
*C. albicans* augmented beta-lactam and vancomycin-resistant gene expression in *S. aureus*. (**a**) Heat map of differentially expressed staphylococcal genes related to drug resistance (*n* = 3). (**b**) Relative expression of genes related to antibiotic resistance in *S. aureus* or co-cultured *S. aureus* measured by RT-qPCR (*n* = 3). All gene expression levels were normalized by 16 s rRNA gene expression. *: *p* < 0.05; **: *p* < 0.01; ***: *p* < 0.001.

Interestingly, the pathways in “Vancomycin resistance” as well as “Beta-Lactam resistance” were also enriched in the KEGG pathway analysis (Figure S2). We then checked the genes related to the cell wall biosynthesis and drug resistance, including *glmU*, *murC* and *murD* as well as the direct target of beta-lactams and penicillin-binding proteins (SAOUHSC_01467, SAOUHSC_01145, SAOUHSC_01652 and SAOUHSC_00646). As expected, these genes were elevated in the co-cultured samples (Figure 4a), and the RT-qPCR analysis confirmed their expression (Figure 4b).

### 2.5. Fungal Ergosterol Biosynthesis and Drug Transmembrane Transportation Were Up-Regulated by S. aureus

**Figure 5:**
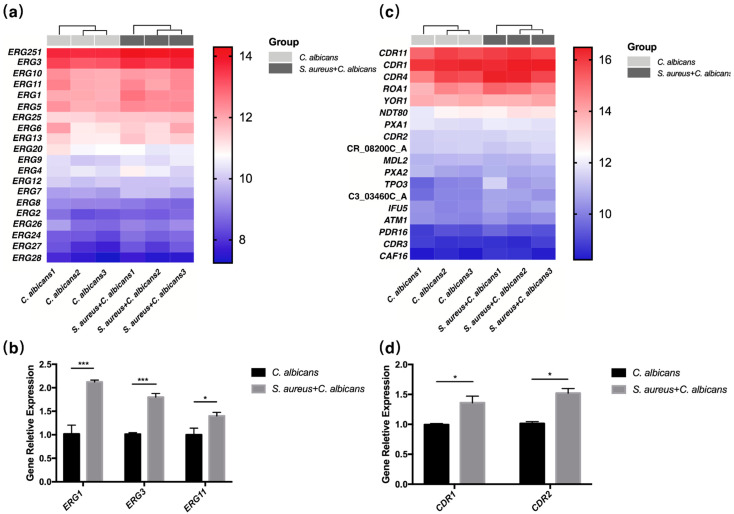
Fungal ergosterol biosynthesis and drug transmembrane transportation were up-regulated by *S. aureus*. (**a**) Heat map of differentially expressed fungal ergosterol biosynthesis genes (*n* = 3). (**b**) Relative expression of selected ergosterol biosynthesis genes in *C. albicans* or co-cultured *C. albicans* measured by RT-qPCR (*n* = 3). All gene expression levels were normalized by 18 s rRNA gene expression. (**c**) Heat map of differentially expressed fungal genes related to drug transmembrane transportation (*n* = 3). (**d**) Relative expression of drug transmembrane transportation genes in *C. albicans* or co-cultured *C. albicans* measured by RT-qPCR (*n* = 3). All gene expression levels were normalized by 18 s rRNA gene expression. *: *p* < 0.05; ***: *p* < 0.001.

Compared to the transcriptomic changes in *S. aureus*, the pathways “Drug transmembrane transporter activity” and “Steroid biosynthesis” of *C. albicans* were also significantly changed (Figures S4 and S5), indicating an increase in the drug-resistant abilities of *C. albicans* in co-cultures. We then found that ergosterol biosynthesis genes and drug-resistance transporter genes were significantly up-regulated (Figure 5a,c), and their expressions were also confirmed by RT-qPCR analysis (Figure 5b,d).

### 2.6. C. albicans Increased the Drug Resistance of S. aureus In Vitro as Well as in Cutaneous Abscess and Peritonitis Murine Models

**Figure 6:**
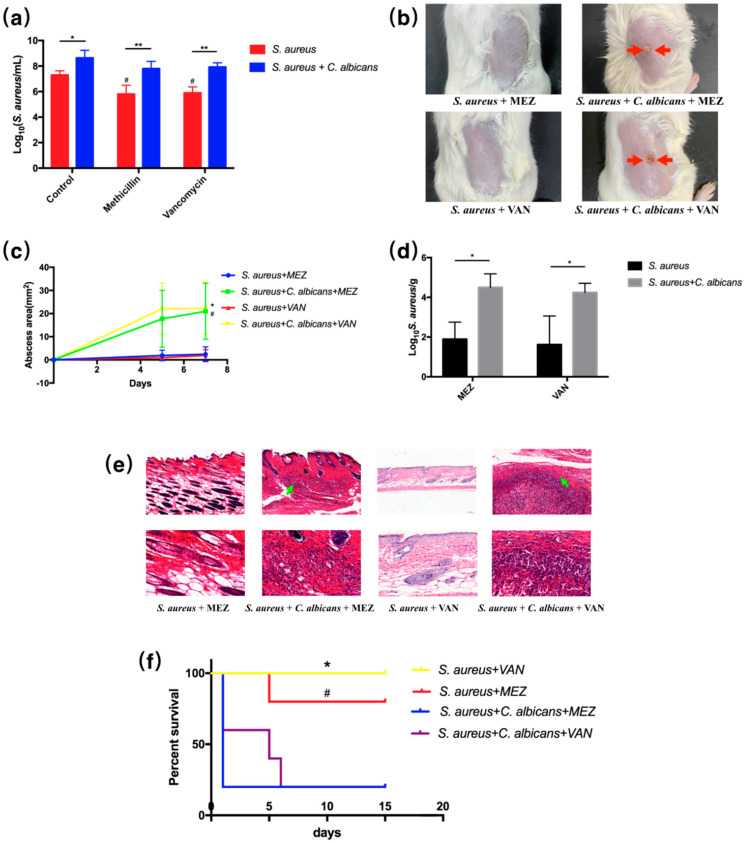
*C. albicans* increased the drug resistance of *S. aureus* in vitro as well as in cutaneous abscess and peritonitis murine models. (**a**) Antimicrobial assay of the single- and dual-species biofilms quantified by CFU (*n* = 3); *: *p* < 0.05; **: *p* < 0.01; #: *p* < 0.05 compared to the control *S. aureus* group. (**b**) Images of infected mice skin after 7 day inoculation of microbial cultures and intervention with antibiotics. Cutaneous abscess is indicated by red arrows. (**c**) Abscess area measured at day 0, 5 and 7 (*n* = 4); *: *p* < 0.05 compared to the *S. aureus* + VAN group; #: *p* < 0.05 compared to the *S. aureus* + MEZ group. (**d**) Staphylcococcal burdens obtained from the infected skin after 7 day inoculation of *C. albicans* and *S. aureus* and intervention with antibiotics (*n* = 3); *: *p* < 0.05. (**e**) Representative histological images mice skin. Necrosis is indicated by green arrows. (**f**) Survival curves of mice intraperitoneally infected with germs and intervention with antibiotics (*n* = 5); *: *p* < 0.05 compared to the *S. aureus* + *C. albicans* + VAN group; #: *p* < 0.05 compared to the *S. aureus* + *C. albicans* + MEZ group.

To demonstrate the synergistic effect of *C. albicans* and *S. aureus* on drug resistance, which we observed from the transcriptome analysis, two commonly used antibiotics to combat *S. aureus*—methicillin (MEZ) and vancomycin (VAN)—were selected for antimicrobial assays both in vitro and *in vivo*. We used 25-fold MIC of MEZ and 50-fold MIC of VAN to treat biofilms in vitro (Table S1). MEZ and VAN both showed significant bactericidal effect in the single *S. aureus* group, while no significant difference was observed between the dual-species control group and the dual-species treated groups. Bacterial CFUs recovered from the MEZ and VAN treated biofilms significantly increased in the combinational groups (Figure 6a), indicating an increase in drug resistance to both antibacterial agents of the *S. aureus* and *C. albicans* combination. In the cutaneous abscess murine model, superficial dermonecrosis or subcutaneous abscess was rarely seen in MEZ and VAN treated single-species groups; however, MEZ and VAN failed to treat infections in the dual-species infected mice (Figure 6b), as the dual-species treated mice, even with antibiotic intervention, displayed increased abscess area and burdens of *S. aureus* (Figure 6c,d). Histological analysis indicated that skin necrosis was found only in dual-species groups treated by antibacterial agents (Figure 6e). In the peritonitis model, MEZ and VAN treatments significantly increased the survival rates of the mice infected by *S. aureus* alone; however, the mice infected by the combination of *S. aureus* and *C. albicans* significantly decreased the survival rates of mice treated by MEZ and by VAN (Figure 6f).

### 2.7. S. aureus Elevated the Drug Resistance of C. albicans In Vitro as Well as in Cutaneous Abscess and Peritonitis Murine Models

**Figure 7:**
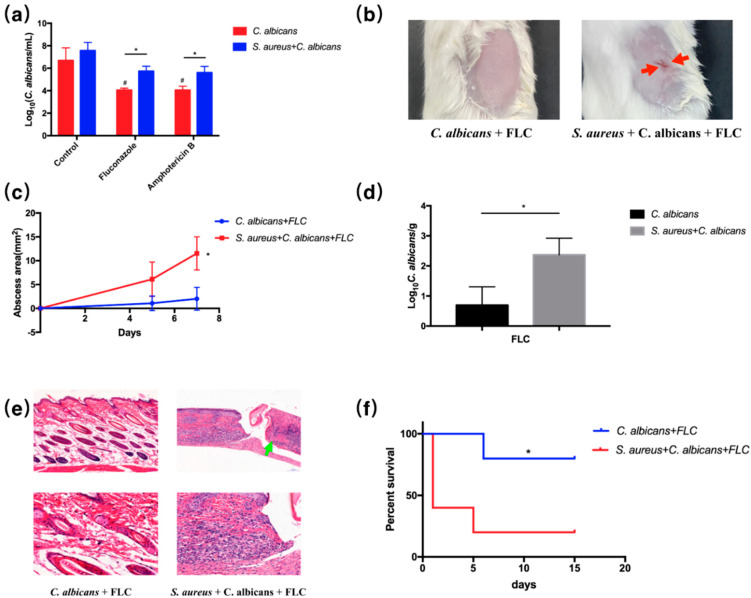
*S. aureus* elevated the drug resistance of *C. albicans* in vitro as well as in cutaneous abscess and peritonitis murine models. (**a**) Antimicrobial assay of the single- and dual-species biofilms quantified by CFU (*n* = 3); *: *p* < 0.05, #: *p* < 0.001 compared to the control *C. albicans* group. (**b**) Images of infected mice skin after 7 day inoculation of microbial cultures and intervention with antibiotics. Cutaneous abscess is indicated by red arrows. (**c**) Abscess area measured at day 0, 5 and 7 (*n* = 4); *: *p* < 0.05. (**d**) Fungal burdens obtained from the infected skin after 7 day inoculation of *C. albicans* and *S. aureus* and intervention with antibiotics (*n* = 3); *: *p* < 0.05. (**e**) Representative histological images mice skin. Necrosis is indicated by green arrows. (**f**) Survival curves of mice intraperitoneally infected with germs and intervention with antibiotics (*n* = 5); *: *p* < 0.05.

Similarly, two commonly used antifungal drugs—fluconazole (FLC) and Amphotericin B (AmB)—were used for anti-fungal assays. We used five-fold MIC of FLC and five-fold MIC of AmB to treat the biofilms in vitro (Table S1). FLC and AmB both showed significant fungicidal effect in the single *C. albicans* group, and fungal CFUs recovered from the FLC and AmB treated biofilms were significantly increased in the combinational groups (Figure 7a), indicating increased drug resistance of the *S. aureus* and *C. albicans* combination. Superficial dermonecrosis and subcutaneous abscess were rarely seen in FLC-treated single-species groups; however, they were commonly seen in dual-species infected mice (Figure 7b), as the dual-species treated mice, even with antibiotic intervention, displayed increased abscess area and burdens of *C. albicans* (Figure 7c,d). Histological analysis indicated that skin necrosis was found only in dual-species groups treated by antifungal agents (Figure 7e). Additionally, FLC treatment increased survival rates of the mice infected by *C. albicans* alone in the peritonitis model; however, the mice infected by the combination of *S. aureus* and *C. albicans* had significantly decreased survival rates, even when treated by FLC (Figure 7f).

## 3. Discussion

Cross-kingdom interactions between species can impact pathogenic behaviors such as proliferation, virulence, and antibiotic tolerance [1,2,3,4]. Figuring out these interactions is essential for identifying new medicinal targets and controlling infectious diseases.

In this study, we confirmed the synergistic, pathogenic and drug-resistant effects of *S. aureus* and *C. albicans* both in vitro and *in vivo*. *C. albicans* was shown to activate *S. aureus* proliferation and augment its virulence and resistance to methicillin as well as vancomycin. On the other hand, *S. aureus* also promoted fungal pathogenicity and azole resistance. Inoculation of both organisms significantly increased mice mortality and skin lesion size, and the curative effect of the antibiotic was dramatically reduced (Figure 1, Figure 2, Figure 6 and Figure 7).

CAF2-1 was chosen for the wild-type strain rather than SC5314, because one of the tasks of our project was to identify the critical signalling pathways from *C. albicans*, which can regulate its cross-kingdom interactions with *S. aureus*, and multiple mutants constructed from CAF2-1 have been screened (unpublished). In addition, no significant difference was observed between CAF2-1 and SC5314 in our previous experiments in terms of synergy with *S. aureus*.

RPMI 1640 medium was employed based on previous research [17,23,33,34]. RPMI 1640, as a cell culture medium, was able to simulate the human body environment and was nutritionally sufficient for the growth of both strains. We validated the synergy between *C. albicans* and *S. aureus* in YPD medium, which did not induce the filamentation of *C. albicans* in our previous experiments. We found that there was no synergy in YPD medium because *S. aureus* could not grow well in this medium. Meanwhile, some of the *C. albicans* mutants in yeast form have also been shown to synergize with *S. aureus* (unpublished), indicating that the synergy may not depend on the hyphae of *C. albicans*.

Fluconazole and amphotericin B, rather than caspofungin, were employed because caspofungin works by noncompetitive inhibition of the enzyme beta-(1,3)-D-glucan synthase, which is a critical component of the biosynthesis of the fungal cell wall. According to our transcriptomic analysis, the genes of *C. albicans* related to azole and polyene resistance were mostly increased, but the target gene of caspofungin was not affected. Therefore, fluconazole and amphotericin B were employed for further assays to confirm the enhanced drug resistance in the dual-species biofilm. However, we believe caspofungin would serve as a good candidate for blocking the synergism of *S. aureus* and *C. albicans* and treating the co-infection. The effects of caspofungin should be validated in future studies.

In cutaneous abscess and peritonitis models, dual-species infected mice showed weak response to the anti-microbial agent treatment. We believe that this can be attributed to both the increased drug resistance and the virulence of the *S. aureus* and *C. albicans* combination. The bacterial and fungal burdens recovered from the murine skin were significantly increased, even under the anti-bacterial and anti-fungal treatment, indicating drug resistance *in vivo*. Additionally, we employed high concentrations (at least five-fold MIC) of drugs to treat the biofilms *in vitro*, and the microbial viability was also increased in the dual-species biofilm. Meanwhile, the virulence factors of *S. aureus* from the dual-species biofilm were significantly increased according to our transcriptome analysis, the qRT-PCR analysis and the in vivo results, as shown in Figure 2. Therefore, both increased drug resistance and virulence contributed to the pathogenicity of the dual-species infections.

In addition, we believe that the dual-species infection could increase the drug resistance and reduce the curative effect of antibiotics; however, antibiotics could also inhibit small amounts of pathogens, so the abscess area induced by the two treated germs is smaller than that induced by the untreated germs. We did not find references to determine the MIC or killing curves of one antibiotic in a dual-species culture, and to our knowledge, the other microorganism would still survive under one antibiotic treatment. In our case, for example, the methicillin had no effects on *C. albicans*, and we could not measure or define the MIC of the dual-species culture. To validate the capability of the microorganism against antibiotics, we employed a high dosage of antibiotics/antifungals to treat the biofilm and determine the viability of each microorganism according to different references, which indicated that this method was commonly used to test the antibiotic effectiveness in a polymicrobial biofilm [24,35,36]. In particular, we used 25-fold MIC methicillin, 50-fold MIC vancomycin, 5-fold MIC fluconazole and 5-fold MIC amphotericin B to treat the dual-species culture. We found that both *S. aureus* and *C. albicans* in the dual-species culture can survive under high dosages of antibiotics or antifungals, suggesting their resistance to the antimicrobial agents in the dual-species culture.

The increase of CFU in the dual-species antibiotic-treated group was not due to the differential growth rate, because a high dosage of antibiotics (25-fold methicillin and 50-fold vancomycin) could significantly reduce the CFU of *S. aureus* in its single-species biofilm (red columns in Figure 6a), but no effect was observed between the dual-species control and antibiotic-treatment groups (blue columns in Figure 6a). As seen in Figure 7a, antifungal agents also significantly reduced the CFU counts of *C. albicans*, while *S. aureus* was not able to promote the growth rate of *C. albicans*, as specified in previous research [17,28]. Meanwhile, the application of antibiotics is commonly thought to affect the growth rate of microorganisms, and the change in growth rate has been observed in antibiotic-resistant cells [37,38]. The growth rates of microorganisms with or without antibiotic treatment are not directly comparable. Therefore, combining the in vitro result with the transcriptomic and qPCR analysis, we believe that there is a synergistic effect in drug resistance between *S. aureus* and *C. albicans*.

A similar increase in CFUs was observed in dual-species treated mice compared with the non-treated mice. However, a high dosage of antibiotics/antifungals reduced at least 10-fold the bacterial/fungal burdens in mice. Besides, growth rates are not directly comparable under the condition of antibiotic treatment as above mentioned. The application of antibacterial or antifungal agents could easily cure the infections caused by a single species of *S. aureus* or *C. albicans*. However, these drugs failed to treat infections in the dual-species infected mice. Thus, we believe the synergistic effect in drug resistance between *S. aureus* and *C. albicans* can also be observed *in vivo*.

Moreover, the mechanisms behind this synergistic effect are still not clear. The accessory gene regulator (agr) quorum-sensing system is well characterized for governing the virulence factors of *S. aureus* [39]. Several studies have demonstrated that *S. aureus* virulence might be augmented by *C. albicans* through this system [30,31]. Nevertheless, in our study, the agr system was down-regulated in the *S. aureus* and *C. albicans* co-cultured group, while an increase in toxin expression was still observed, which may indicate another pathway managing staphylococcal pathogenicity. A recent study showed that high levels of glycometabolism, leading to increased intracellular ATP, might up-regulate the agr system activity and subsequently promote *S. aureus* infection [40]. Interestingly, our transcriptome analysis also revealed an elevation in sugar transmembrane transportation and metabolism, which may explain the increasingly expressed virulence factors (Figure S6). Additionally, despite the traditional viewpoint that *S. aureus* adherence to *C. albicans* hyphae results in rising pathogenicity [41], the impact of *S. aureus* cast on *C. albicans* was also studied. Ergosterol, the most important component in the fungal cell membrane, is associated with coordinating membrane heterogeneity, preventing water penetration, and maintaining the integrity, rigidity and fluidity of the plasma membrane [42,43,44]. Ergosterol not only serves as the basis of fungal growth virulence but also acts as the direct target of azoles [45]. Four major mechanisms are characterized to generate resistance to azoles in the *C. albicans* ergosterol biosynthetic pathway, including the up-regulation or mutations of the *ERG11* gene [46,47]. Therefore, our findings indicating the activation of the ergosterol biosynthetic pathway may provide a novel clue for the synergistic pathogenicity and azole resistance in *C. albicans* and *S. aureus* dual-species cultures (Figure 5a,b and Figure S4). For the inducing vancomycin resistance in *S. aureus*, most scholars believe that the biofilm matrix provides the most important contribution. It has been demonstrated that *S. aureus* may be coated in the matrix secreted by *C. albicans*, and the *C. albicans*-secreted β-1,3-glucan cell wall component has been identified as the key matrix constituent providing the bacterium with enhanced drug tolerance [24,27,32]. However, in the present study, the staphylococcal transcriptome was also adjusted by *C. albicans*. Most drugs used for controlling *S. aureus*-related infections target cell wall biosynthesis, and increased peptidoglycan cross-linking is believed to contribute to the acquirement of drug-resistant capability. It has been observed that decreased peptidoglycan cross-linking leads to increased susceptibility toward cell-wall-targeting antibiotics, namely β-lactams and vancomycin [48,49]. Our transcriptome results, suggesting the over-expression of cell wall biosynthetic pathways, including penicillin-binding proteins, imply another way for the up-regulation of antibiotic resistance in *S. aureus* and *C. albicans* dual-species biofilms (Figure 4a,b and Figure S2). In addition, despite the above-mentioned ergosterol biosynthetic pathway, drug-efflux pumps were also augmented in *C. albicans* when co-cultured with *S. aureus*, indicating another way *S. aureus* may participate in *C. albicans* drug resistance (Figure 5c,d and Figure S5).

In conclusion, this study confirmed the synergistic effect between *S. aureus* and *C. albicans* on pathogenicity and drug resistance. The mechanisms behind this effect were explored by transcriptome analysis, which revealed up-regulation in both virulence factors and drug-resistant pathways in *S. aureus* and *C. albicans*, providing us with a novel view for determining the cross-kingdom interactions between species and for discovering new medicines to combat multiple-species-associated infections. The synergistic interactions between *S. aureus* and *C. albicans* on pathogenesis and drug resistance highlight the importance of targeting the microbial interactions in polyspecies-associated infections.

## 4. Materials and Methods

### 4.1. Chemicals

Fluconazole (J&K, Shanghai, China) and Amphotericin B (AMRESCO, Solon, OH, USA) were dissolved with dimethyl sulfoxide (DMSO, Merck-China, Chengdu, China) and stored at −20 °C until use. Vancomycin hydrochloride (Solarbio, Beijing, China) and methicillin (APExbio, Shanghai, China) were dissolved with deionized water and stored at −20 °C until use.

### 4.2. Strains and Cultivation

*C. albicans* wild-type strain CAF2-1 (*ura3Δ::imm434/URA3 iro1Δ::imm434/IRO1*, parent: SC5314) [50] and *S. aureus* NCTC 8325-4 were used in this study. The green fluorescent protein (GFP) expressing *S. aureus* was provided by Prof. Baolin Sun (University of Science and Technology of China) [51,52,53]. Organisms were stored as frozen stocks at −80 °C. *C. albicans* was maintained on YPD plates (1% yeast extract, 2% peptone, 2% glucose and 2% agar), while *S. aureus* was maintained on TSB plates (3% trypticase soy broth and 2% agar). The colonies of *C. albicans* and *S. aureus* were picked out and placed into liquid YPD medium or TSB medium and incubated at 35 °C and 37 °C overnight, respectively. For the GFP-expressing *S. aureus*, 15 μg/mL of chloramphenicol was added for fluorescence detection [53]. Cells were harvested by centrifugation at 5000 rpm, 4 °C for 10 min, followed by washing in PBS three times. Then, both of the final suspensions were adjusted to 1 × 10^6^ CFU/mL concentration in RPMI 1640 (Solarbio, Beijing, China) supplemented with L-glutamine and 5% fetal bovine serum (FBS, Gibco, Grand Island, NY, USA) for co-culture at 37 °C (*n* = 7).

### 4.3. Scanning Electron Microscopy (SEM)

The single-species or dual-species biofilms were grown on glass coverslips in RPMI 1640 supplemented with L-glutamine and 5% fetal bovine serum at 37 °C, with both organisms adjusted to a final concentration of 1 × 10^6^ CFU/mL. Both germs were added at the same time. After 24 h, the medium was gently aspirated, and non-adherent cells were removed by washing in PBS three times. Samples were then fixed overnight at 4 °C with 4% paraformaldehyde, followed by dehydration through a graded ethanol series [54] and sputter-coating with gold. Biofilm images were recorded using scanning electron microscopy (FEI, Hillsboro, OR, USA).

### 4.4. Crystal Violet Staining

Biofilms were grown in RPMI 1640 for 24 h as above described (*n* = 5). Single-species or dual-species biofilms were stained with crystal violet (CV) solution as described previously [55]. In brief, biofilms cultured in 96-well plates were fixed with methanol. At total of 0.2% crystal violet solution was then added to each well and incubated for 20 min at room temperature. Excess CV solution was removed by washing with deionized water, and bounded CV was released by 33% acetic acid. Absorbance was detected at 570 nm.

### 4.5. Antimicrobial Assay

The minimum inhibitory concentration (MIC) of methicillin, vancomycin, fluconazole and amphotericin B was determined in accordance with a broth microdilution protocol modified from the Clinical and Laboratory Standards Institute M07-A9 methods (National Committee for Clinical Laboratory Standards, 2012). The single-species or dual-species biofilms were grown as described above (*n* = 3). After 24 h, the medium was gently aspirated, and non-adherent cells were removed by washing in PBS three times. Methicillin (25 μg/mL) and vancomycin (25 μg/mL) were added to the single *S. aureus* biofilm or the dual-species biofilm, respectively, while fluconazole (2.5 μg/mL) and amphotericin B (10 μg/mL) were added to both the single *C. albicans* biofilm or the dual-species biofilm separately. Plates were incubated for an additional 24 h. Fungal and bacterial viability was monitored by CFU assays on CHROMagar^TM^ Candida (France) plates for *C. albicans* and on mannitol salt agar (Qingdao Hope Bio-Technology Co., Ltd., Qingdao, China) plates for *S. aureus*.

### 4.6. Murine Models

All animal works were conducted in strict accordance with the guidelines of the Ethics Committee of West China School of Sichuan University, and the protocols were fully approved by this Agency (license number WCHSIRB-D-2020-406).

The murine peritonitis and cutaneous abscess models were performed according to previous descriptions [56,57,58]. For the peritonitis model, female ICR (CD-1) mice weighing between 22 g and 25 g were inoculated intraperitoneally with a dose of *C. albicans* (1 × 10^7^ CFUs in total) or *S. aureus* (1 × 10^8^ CFUs in total) or both organisms suspended in 0.9% NaCl solution (*n* = 5). Antimicrobial interventions were performed by i.p. injection with methicillin (25 mg/kg), vancomycin (25 mg/kg) or fluconazole (10 mg/kg) at 0 h, 24 h and 48 h. Mice mortality was monitored for 15 days. For the cutaneous abscess model, mice were shaved of flank fur and then subcutaneously injected with *S. aureus* (1 × 10^7^ CFUs in total) or *C. albicans* (1 × 10^6^ CFUs in total) or both organisms into the shaved area (*n* = 6). Antimicrobial interventions were performed with a pocket injection of methicillin (5 mg/kg), vancomycin (5 mg/kg) or fluconazole (2.5 mg/kg) at 0 h. Abscess areas were monitored and calculated according to the formula: π × (L/2) × (W/2). After 7 days, mice were sacrificed, and CFUs were counted. An infected skin sample was randomly selected from each group for the hematoxylin and eosin stain.

### 4.7. Transcriptome Analysis

Total RNA of 24 h single-species cultures and dual-species cultures was extracted, and cDNA libraries were prepared as previously described (*n* = 3) [59]. The cDNA was shotgun sequenced (101 bp paired-end reads) with an Illumina HiSeq 4000 instrument (Illumina, San Diego, CA, USA) using a customer sequencing service (Majorbio Co., Ltd., Shanghai, China). Sequencing reads were statistically analyzed, and quality was assessed by FASTQC (http://www.bioinformatics.babraham.ac.uk/projects/fastqc/, accessed date: 28 February 2018) and then processed by Trimmomatic to remove adapter sequences and low-quality reads with average quality scores lower than 15. Reads that were less than 50 base pairs (bp) after trimming were also excluded from further genome mapping [60,61,62,63]. Transcriptome analysis, including data processing, mapping of reads, differentially expressed genes (DEGs) and enrichment analysis, was performed according to previous instructions [64,65,66]. R statistical package software DESeq2 (http://bioconductor.org/packages/stats/bioc/DESeq2/, accessed date: 25 November 2018) was utilized for differential expression analysis. Cluster analysis was carried out based on the heat map, and the results were drawn as tree maps (pedigree maps) in the heat map. Gene Ontology (GO) annotation was conducted through the Blast2GO software, and the Kyoto Encyclopedia of Genes and Genomes (KEGG) was used for metabolic pathway analysis through the KOBAS2.0 software [60,61,62,63]. The sequencing data from this study have been submitted (https://www.ncbi.nlm.nih.gov/sra/PRJNA700494) to NCBI’s Sequence Read Archive under accession no. PRJNA700494.

### 4.8. Relative Quantification of Differentially Expressed Genes by RT-qPCR

*S. aureus* or *C. albicans* or both organisms were cultured in RPMI 1640 at 37 °C for 24 h as described in Section 4.2. Cells were collected by centrifugation at 5000 r/min, at 4 °C for 10 min (*n* = 3). Total RNA was extracted with 1 mL TRIZol reagent (Invitrogen, Carlsbad, CA, USA) following the manufacturer’s instructions. cDNA was synthesized according to the One Step RNA PCR kit (Takara Inc., Chengdu, China) protocols. The RT-qPCR was then performed following the SYBR^®^ PremixEx Taq^TM^ kit (Takara Inc., Chengdu, China) two-step strategy: (1) 95 °C for 30 s; (2) 40 PCR cycles (95 °C for 5 s, a gene-specific annealing temperature for 30 s). All primer sequences used are listed in Table S2. RT-qPCRs were run on LightCycler 480 II (Roche, Basel, Switzerland). The gene expression level relative to the calibrator was expressed as 2^−ΔΔCT^.

### 4.9. Statistical Analysis

The data were expressed as the mean ± standard deviation (mean ± SD). Shapiro–Wilk normality tests were applied to determine whether data conformed to a normal distribution. Statistical significance was decided by Student’s *t*-test with Welch’s correction, one-way ANOVA with Dunnett’s or Tukey’s multiple comparison test, two-way ANOVA with Sidak’s multiple comparisons test or Log-rank (Mantel–Cox) test. *p* < 0.05 was defined as statistically significant, and *p* < 0.01 was considered highly significant. All statistical analyses of the data were performed using GraphPad Prism 7 software.

## Data Availability

The sequencing data from this study have been submitted (https://www.ncbi.nlm.nih.gov/sra/PRJNA700494) to NCBI’s Sequence Read Archive under accession no. PRJNA700494.

## Supplementary Materials

The following are available online at https://www.mdpi.com/article/10.3390/pathogens10081036/s1, Table S1: The minimum inhibitory concentration; Table S2: Specific primers used for RT-qPCR; Figure S1: GFP expressing *S. aureus* was co-cultured with *C. albicans*. GFP fluorescence intensity was detected at 14 h (*n* = 7). ***: p < 0.001; Figure S2: KEGG pathway enrichment of differentially expressed staphylococcal genes. Pathways related to staphylococcal pathogenicity and antibiotic resistance are indicated by red arrows; Figure S3: Gene ontology terms of differentially expressed staphylococcal genes. The term associated with staphylococcal pathogenicity is indicated by a red arrow; Figure S4: KEGG pathway analysis of differentially expressed fungal genes. The fungal pathway related to ergosterol biosynthesis is indicated by a red arrow; Figure S5: Gene ontology terms of differentially expressed fungal genes. The term associated with drug transmembrane transporter activity is indicated by a red arrow; Figure S6: Heat map of differentially expressed staphylococcal genes related to sugar transmembrane transportation and metabolism (*n* = 3).
