# Supplementary material for: Staphylococcus aureus Synergized with Candida albicans to Increase the Pathogenesis and Drug Resistance in Cutaneous Abscess and Peritonitis Murine Models
Source: Pathogens. 2021 Aug 16;10(8):1036. doi: 10.3390/pathogens10081036 (PMC8398722; doi:10.3390/pathogens10081036)
Supplement: Supplementary file 1 [file pathogens-10-01036-s001.zip › Supplementary Tables.pdf]

Supplementary Material

Table S1. The minimum inhibitory concentration

Drugs	MIC (µg/mL)	
	S. aureus	*C. albicans*
Methicillin	1	/
Vancomycin	0.5	/
Fluconazole	/	0.5
Amphotericin B	/	2

Table S2. Specific primers used for RT-qPCR

Primers	Sequences
16s rRNA	Forward: 5’-GTAGGTGGCAAGCGTTAT-3’
	Reverse: 5’-GGTGTTCCTCCATATCTCTG-3’
18s rRNA	Forward: 5’-GAGCCAGCGAGTATAAGC-3’
	Reverse: 5’-GCCTCACTAAGCCATTCAA-3’
SAOUHSC_01121	Forward: 5’-GCAGCAGATAACTTCCTTG-3’
	Reverse: 5’- TGGTAGTCATCACGAACTC-3’
SAOUHSC_02708	Forward: 5’-TGGCTCATTCAACTACTCTA-3’

	Reverse: 5’-GCAGATACTTGACCATTCG-3’
SAOUHSC_00192	Forward: 5’-TGGTACAGGTATCCGTGAA-3’
	Reverse: 5’-TTCGCTTGGCTTCTTGTAT-3’
SAOUHSC_00069	Forward: 5’-CGTAACGGCTTCATCCAA-3’
	Reverse: 5’-CCAGGCTTGTTGTTGTCT-3’
SAOUHSC_01705	Forward: 5’-AAGGTGGCGACATTGTTA-3’
	Reverse: 5’-CCTCACGACCATACTCAC3’
glmU	Forward: 5’-TTGCGGAACGATTACAGT-3’
	Reverse: 5’-TGCCACCAATACATCATCA-3’
SAOUHSC_01467	Forward: 5’-TCTCACCAACACAATTAGCA-3’
	Reverse: 5’-TCACCATCACGAGTAACTAC-3’
murC	Forward: 5’-GGTGATGATGAACATCTACG-3’
	Reverse: 5’-TGTGGAGACAGGAAGTGA-3’
murD	Forward: 5’-CTAATCTCTGAAGCACCAATC-3’
	Reverse: 5’-ATGACGACAACTCTGTAACT-3’
murQ	Forward: 5’-CCGTTCAGTGCGTATTATTC-3’
	Reverse: 5’-GTCTTGTTGCTTCTTCCTTAG-3’
ERG1	Forward: 5’-GGCTGGTATCAAGGCATT-3’
	Reverse: 5’-ACTGGCTTCACTGGATTAG-3’
ERG3	Forward: 5’-CTTGTCACACTGTCCATCA-3’

	Reverse: 5’-GAATCATCTGGTCTTCTGTAAG-3’
ERG11	Forward: 5’-TGGTGGTGGTAGACATAGA-3’
	Reverse: 5’-TCTGCTGGTTCAGTAGGT-3’
CDR1	Forward: 5’-CAACAATACAAGACCAGCAT-3’
	Reverse: 5’-ATAGAACACCGACGACAATA-3’
CDR2	Forward: 5’-ATGTTCTCAAGACGCTTATG-3’
	Reverse: 5’-TGGATTGGTTAAGGAAGTCA-3’
