# Supplementary figures and images for: Staphylococcus aureus Synergized with Candida albicans to Increase the Pathogenesis and Drug Resistance in Cutaneous Abscess and Peritonitis Murine Models
Source: Pathogens. 2021 Aug 16;10(8):1036. doi: 10.3390/pathogens10081036 (PMC8398722; doi:10.3390/pathogens10081036)

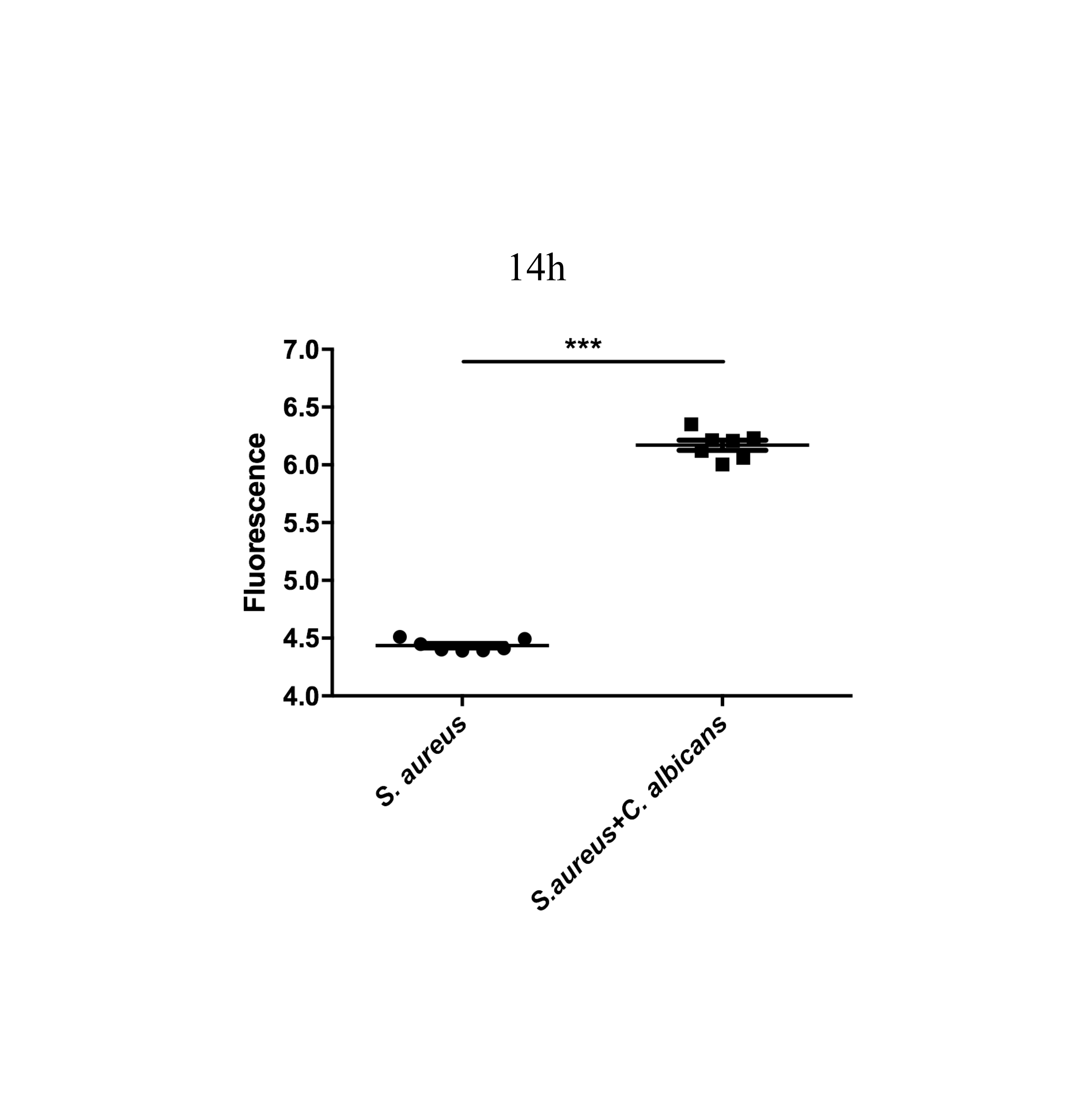

Supplement: Supplementary file 1 [file pathogens-10-01036-s001.zip › Figure S1.tiff]

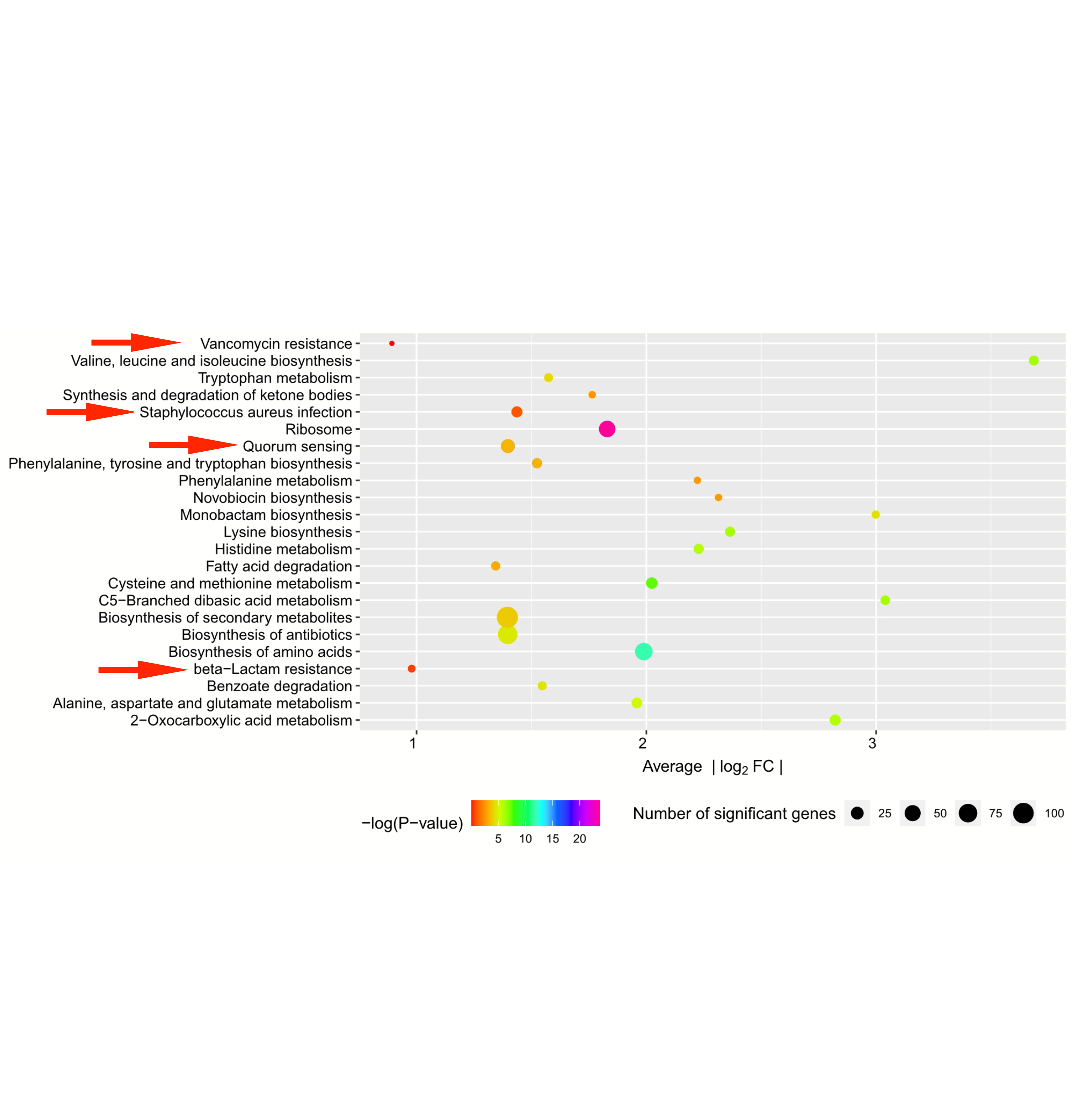

Supplement: Supplementary file 1 [file pathogens-10-01036-s001.zip › Figure S2.tiff]

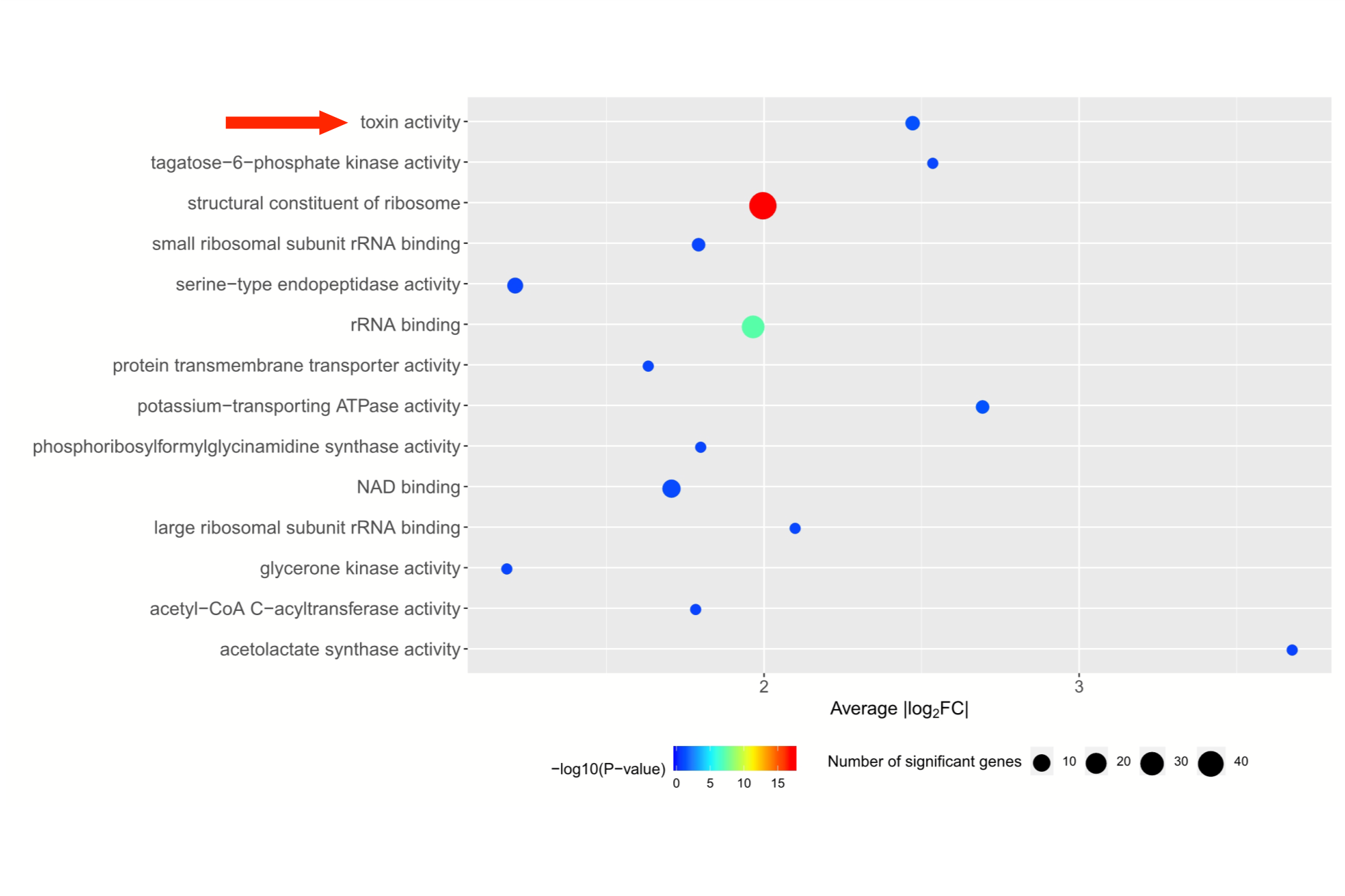

Supplement: Supplementary file 1 [file pathogens-10-01036-s001.zip › Figure S3.tiff]

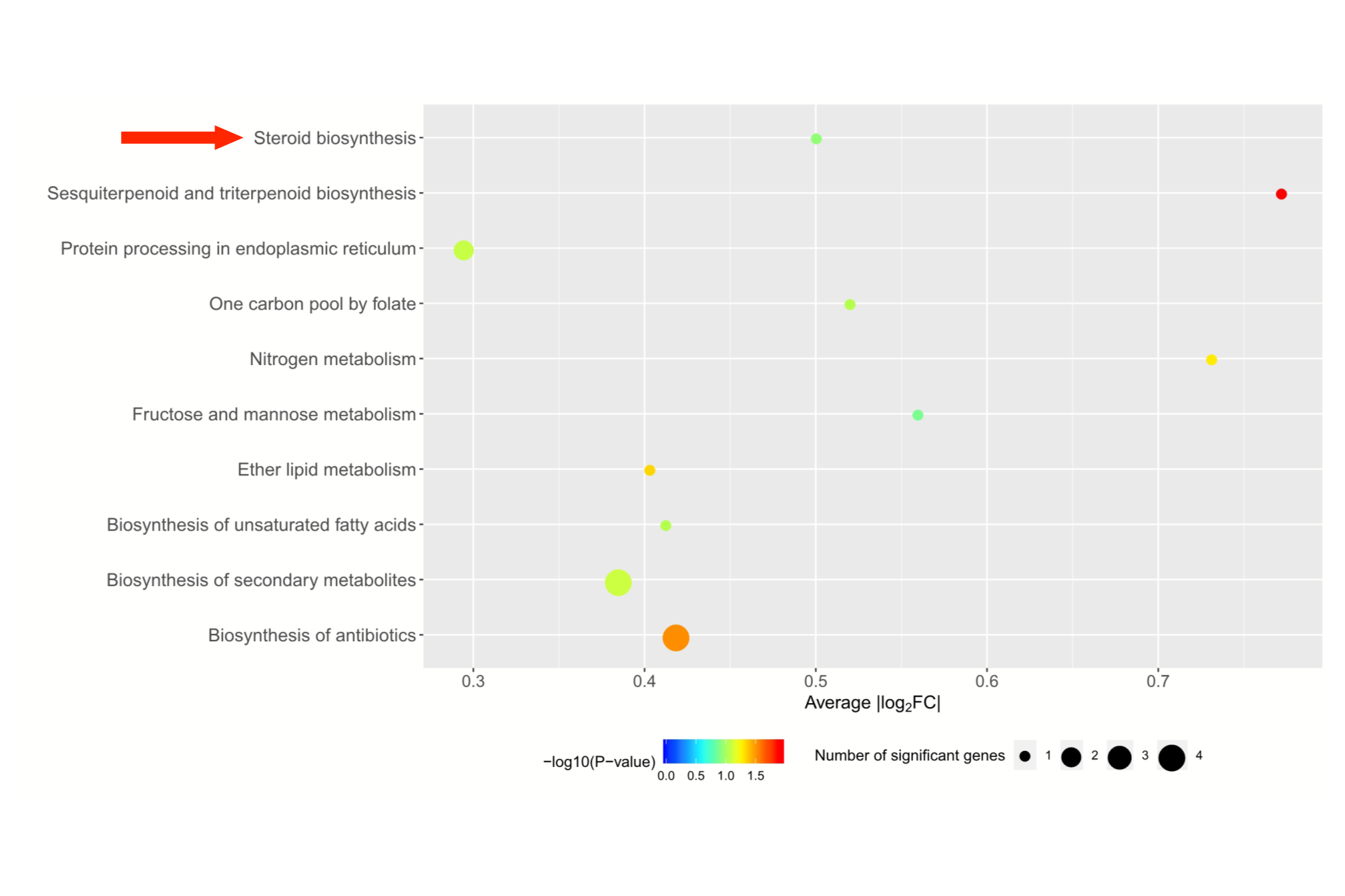

Supplement: Supplementary file 1 [file pathogens-10-01036-s001.zip › Figure S4.tiff]

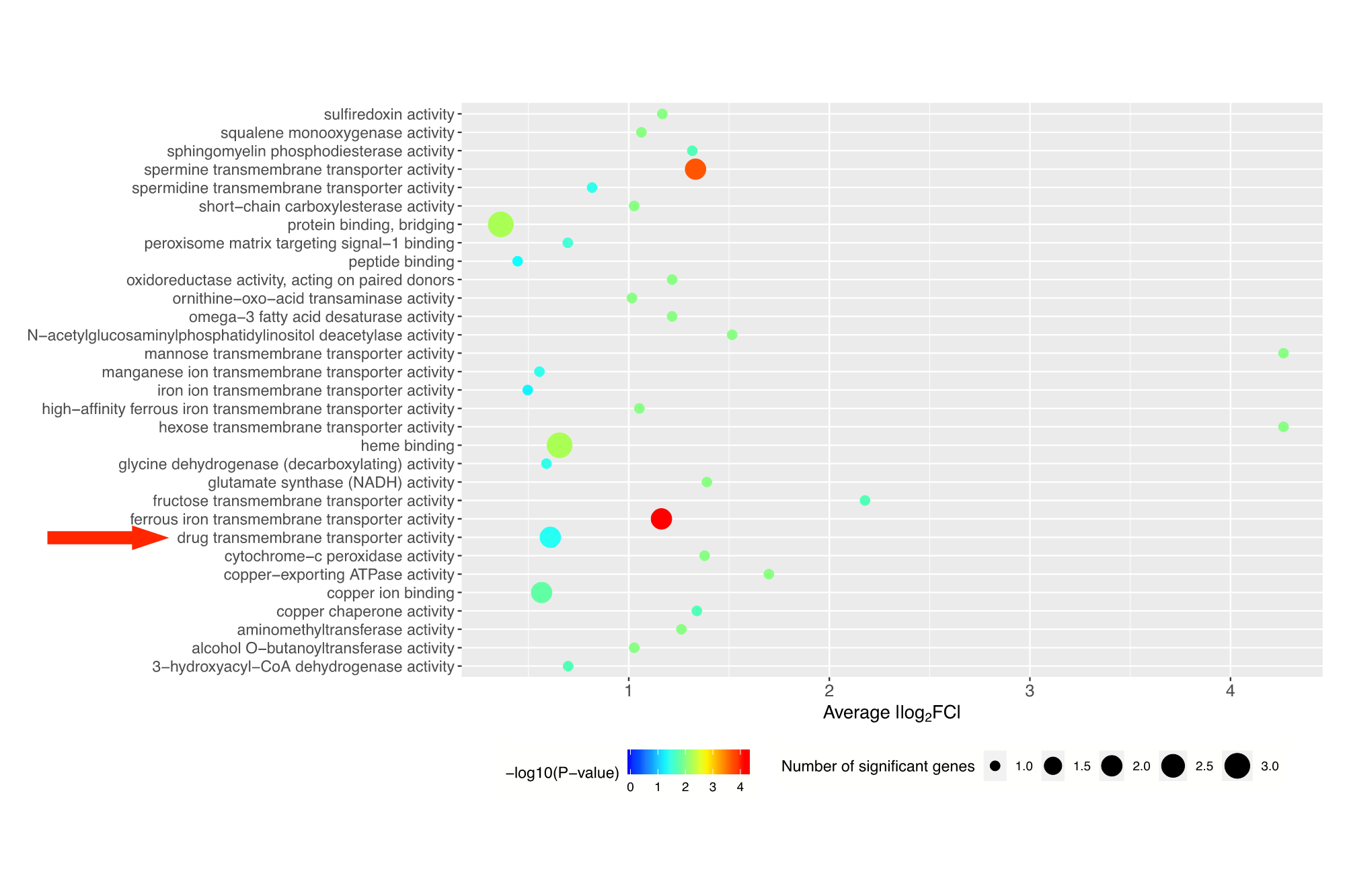

Supplement: Supplementary file 1 [file pathogens-10-01036-s001.zip › Figure S5.tiff]

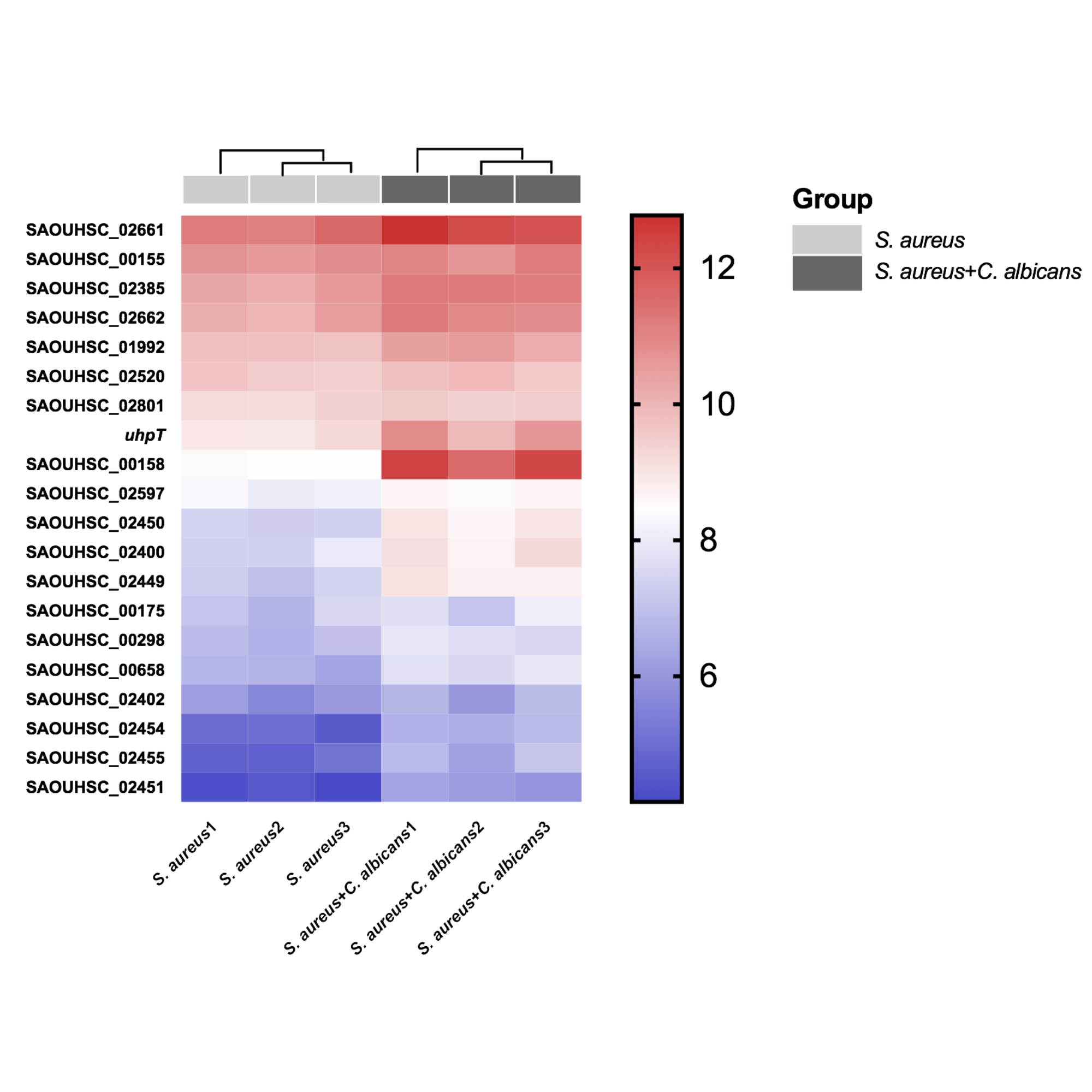

Supplement: Supplementary file 1 [file pathogens-10-01036-s001.zip › Figure S6.tiff]
